# Clinical Islet Transplantation Covered by Health Insurance in Japan

**DOI:** 10.3390/jcm11143977

**Published:** 2022-07-08

**Authors:** Hirofumi Noguchi

**Affiliations:** Department of Regenerative Medicine, Graduate School of Medicine, University of the Ryukyus, Nishihara 903-0215, Japan; noguchih@med.u-ryukyu.ac.jp; Tel.: +81-98-895-1696; Fax: +81-98-895-1684

Pancreatic islet transplantation is a treatment option for patients with type 1 diabetes mellitus and has been performed in various countries [1,2,3,4,5]. Islet transplantation is a minimally invasive approach for β-cell replacement compared with pancreas transplantation. In Japan, clinical islet transplantation (CIT) has received national health insurance (NHI) coverage since April 2020 [6]. The NHI coverage of CIT is very beneficial to patients with type 1 diabetes who experience hypoglycemic unawareness despite maximal care.

In Japan, a severe donor shortage has been a serious issue. Until 1997, there was no legislation for donation after brain death (DBD) in Japan and, therefore, fewer than 200 cases of kidney transplantation from fewer than 100 donations after circulatory death (DCD) per year were performed [7,8]. In 1997, a law on organ transplantation from DBD donors was enacted, and organ transplantation with DBD started under this law [9]. However, from 1997 to 2009, only a few cases of DBD organ transplantation per year were performed because DBD required the person to make their intention clear before BD.

In 1997, the working group of islet transplantation in the Japanese Pancreas and Islet Transplantation Association (JPITA) officially held a meeting [6]. The working group in JPITA conducted feasibility studies for the implementation of CIT in Japan and defined and standardized the donor criteria for CIT, the recipient criteria, and the facility criteria for islet isolation and CIT [6]. In 2004, we performed the first case of CIT in Japan [10]. The pancreatic graft for the CIT was derived from DCD, without the withdrawal of life-sustaining therapies. CIT has been performed 34 times in 18 cases from 2004 to 2007 in Japan [11,12]; we performed 17 CITs (50% of those in Japan) in this era [5]. Although DBD for CIT was not prohibited in Japan, all transplanted cases were performed with DCD because there were only a few DBD per year from 1997 to 2009 and pancreata with DBD were mainly used for pancreas transplantation at this time. We also performed the first case of CIT using a partial pancreatic graft from a living donor due to the absolute donor shortage in Japan and the patient became insulin-independent [13].

However, the shortage of DBD from 1997 to 2009 remained serious. Therefore, the law on organ transplantation with DBD was amended in 2009 and came into effect in 2010. The main modification of the law was that the declaration of a person’s intent to donate before brain death was no longer required for DBD [6]. After the law was amended, the number of DBD has gradually increased, reaching a record high of 98 cases in 2019 [14], although the number of DBD is still low compared with that in the United States and Europe. As DBD in Japan has increased, the rate of pancreas transplants per DBD has gradually decreased and some pancreata with DBD have been used for CIT from 2013 [14].

In 2007, CIT in Japan was interrupted because it was discovered that bovine brain component was used in the manufacturing process of collagenase for islet isolation [15]. In 2013, CIT in Japan was reopened using pancreata with DBD because of the increase in DBD from 2010. We performed the first case of CIT with DBD in Japan [6]. CIT has been performed 18 times in nine cases from 2013 to 2020 in Japan [6]; we performed CIT 15 times in this period (83% of such transplantations in Japan). The proportion of patients with HbA_1c_ of <7.4% without severe hypoglycemic attacks from 90 days to 365 days after the first CIT made up 75% of cases. Islet graft survival (C-peptide level: >0.3 ng/mL) at 4 years after the first islet infusion was achieved in 80% of the cases [6]. Based on these results, CIT has received NHI coverage from April 2020 [6].

In Japan, we performed 21 clinical islet isolations and 17 CITs with DCD from 2004 to 2007 and 16 clinical islet isolations and 15 CITs with DBD from 2013 to 2020. The high success rate of CIT with not only DBD (94%) but also DCD (81%) is due to our modifications of the Ricordi/Edmonton islet isolation methods. These modifications included pancreatic ductal injection of a preservation solution [16,17], pancreas preservation with MK solution [18], and the use of an iodixanol-based purification solution [17,19,20,21] and islet culture/preservation [22,23,24]. The islet isolation technique also enabled us to perform successful single-donor CITs with DBD from 2007 to 2010 in the United States [25].

The number of islets from one donor pancreas is usually insufficient to achieve insulin independence [1,26,27,28], although we achieved it from a single donor in the United States [25]. The islet isolation procedure destroys the cellular and non-cellular components of the pancreas and the activation of some components, including resident neutrophils, macrophages, and T cells, probably plays an important role in the impairment of islet survival [26,27,28,29,30]. Although the NHI coverage of allogeneic CIT in Japan is a turning point, the donor shortage in Japan is still serious. The improvement of the islet isolation technique as well as an increase in DBD are key factors of successful CIT in Japan.
